# Relapse patterns and outcome after relapse in standard risk medulloblastoma: a report from the HIT-SIOP-PNET4 study

**DOI:** 10.1007/s11060-016-2202-1

**Published:** 2016-07-16

**Authors:** Magnus Sabel, Gudrun Fleischhack, Stephan Tippelt, Göran Gustafsson, François Doz, Rolf Kortmann, Maura Massimino, Aurora Navajas, Katja von Hoff, Stefan Rutkowski, Monika Warmuth-Metz, Steven C. Clifford, Torsten Pietsch, Barry Pizer, Birgitta Lannering, On behalf of the SIOP-E Brain Tumour Group

**Affiliations:** 1 Department of Paediatrics, University of Gothenburg and Queen Silvia Children’s Hospital, Gothenburg, Sweden; 2 Paediatrics III, Division of Paediatric Haematology and Oncology, University Hospital of Essen, Essen, Germany; 3 Karolinska Institutet, Stockholm, Sweden; 4 Department of Paediatrics, Adolescent and Young Adults Oncology, Institut Curie and University Paris Descartes, Sorbonne Paris Cité, Paris, France; 5 Department of Radiooncology, University Hospital Leipzig, Leipzig, Germany; 6 Fondazione IRCCS Istituto Nazionale dei Tumori, Milan, Italy; 7 Biocruces Health Research Institute, Universidad Del País Vasco/EHU, Bilbao, Spain; 8 University Medical Centre Hamburg-Eppendorf, Hamburg, Germany; 9 University of Würzburg, Würzburg, Germany; 10 Northern Institute for Cancer Research, Newcastle University, Newcastle upon Tyne, UK; 11 Department of Neuropathology, University of Bonn, Bonn, Germany; 12 Alder Hey Children’s Hospital, Liverpool, UK

**Keywords:** Medulloblastoma, Relapse, Survival, Treatment, Clinical trial, Chemotherapy, Radiotherapy, Paediatric, Secondary tumours

## Abstract

The HIT-SIOP-PNET4 randomised trial for standard risk medulloblastoma (MB) (2001–2006) included 338 patients and compared hyperfractionated and conventional radiotherapy. We here report the long-term outcome after a median follow up of 7.8 years, including detailed information on relapse and the treatment of relapse. Data were extracted from the HIT Group Relapsed MB database and by way of a specific case report form. The event-free and overall (OS) survival at 10 years were 76 ± 2 % and 78 ± 2 % respectively with no significant difference between the treatment arms. Seventy-two relapses and three second malignant neoplasms were reported. Thirteen relapses (18 %) were isolated local relapses in the posterior fossa (PF) and 59 (82 %) were craniospinal, metastatic relapses (isolated or multiple) with or without concurrent PF disease. Isolated PF relapse vs all other relapses occurred at mean/median of 38/35 and 28/26 months respectively (p = 0.24). Late relapse, i.e. >5 years from diagnosis, occurred in six patients (8 %). Relapse treatment consisted of combinations of surgery (25 %), focal radiotherapy (RT 22 %), high dose chemotherapy with stem cell rescue (HDSCR 21 %) and conventional chemotherapy (90 %). OS at 5 years after relapse was 6.0 ± 4 %. In multivariate analysis; isolated relapse in PF, and surgery were significantly associated with prolonged survival whereas RT and HDSCR were not. Survival after relapse was not related to biological factors and was very poor despite several patients receiving intensive treatments. Exploration of new drugs is warranted, preferably based on tumour biology from biopsy of the relapsed tumour.

## Introduction

The 5-year progression free survival for medulloblastoma (MB), the most common malignant brain tumour in childhood, is now expected to be 70–80 % in the ‘standard’ or ‘average’ risk subgroup [1]. Between 2001 and 2006, the International Society of Paediatric Oncology (SIOP)-Europe Brain Tumour Group conducted a multicentre Phase III trial, the HIT-SIOP-PNET4 study, for children and young people with ‘standard-risk’ MB, with >100 participating centres. After clinical staging, patients were randomised to receive either conventional/standard radiotherapy (STRT) or hyperfractionated radiotherapy (HFRT) followed by chemotherapy. In addition, tumour biomarkers were examined [2]. We previously reported no significant difference in the probability of event free survival (EFS) between the two treatment groups after a median follow-up of 4.8 years [2]. The outcome after a median follow-up of 7.8 years is included in this report.

In standard risk MB, the major cause of death is due to relapse that occurs in 20−30 % of patients. Several reports have demonstrated that the prognosis at relapse is poor, with generally less than 10 % survival [3–5]. This has caused uncertainty whether intensive second line treatment including re-irradiation or myeloablative chemotherapy is appropriate. In addition, there are increasing reports on the occurrence of second malignant neoplasms (SMN) as a consequence of MB therapy [1]. We therefore describe here the patterns of relapsed disease, treatments received at relapse as chosen by various institutions across Europe, and associated outcomes, for patients enrolled on the HIT-SIOP-PNET4 study.

## Patients and methods

Patients (n = 338) from Germany, France, Italy, UK, Austria, Spain, The Netherlands, Sweden, Norway and Denmark were included in the HIT-SIOP-PNET4 trial. Patients were 4–21 years of age, with a MB without metastases on craniospinal MRI or cerebrospinal fluid (CSF) cytology. All patients were randomised to receive either STRT (1.8 Gy daily) with 23.4 Gy to the craniospinal axis (CSA) and a 30 Gy boost to the posterior fossa (PF) or HFRT (1 Gy twice daily) with 36 Gy CSA, a 24 Gy boost to the PF and another 8 Gy boost to the tumour bed. During RT, a weekly dose of vincristine was given. RT was followed by eight cycles of chemotherapy with cisplatin, vincristine and CCNU, given at 6 week intervals [2]. The study was approved by each national/institutional review board and all patients/parents/guardians had consented to participate.

Until June 30th 2013, when the database was frozen, patients were followed to time of relapse/progression (hereafter called relapse) or SMN as primary event. Time of relapse was defined as the date of radiological examination confirming the relapse. In Germany, MRI scans were centrally reviewed at relapse, in all other countries radiological relapse was diagnosed at the treating centres. For non-German patients, a case report form specifically designed for this analysis was sent to the treating institutions asking for more detailed information on relapse site, symptoms and treatment of relapses and the date of death. For German patients, this information was collected from the HIT Group Relapsed MB database.

At primary diagnosis, tumour material was centrally reviewed by appointed study pathologists, confirming the diagnosis and the histological MB variant. In addition, a series of MB biomarkers were investigated as part of the HIT-SIOP-PNET4 study [6]; (i) β-catenin nuclear accumulation by immunohistochemistry (IHC) and *CTNNB1* exon 3 mutation (DNA sequencing), which both defined the favourable-risk wingless (*WNT*)-activated MB subgroup [6], (ii) *MYC* and *MYCN* gene amplification status (by FISH), associated with a poor prognosis in previous studies [7], and (iii) Chromosome 17 alterations (Chr 17(im)/diploid background; by FISH), which were previously associated with a poor prognosis in the HIT-SIOP-PNET4 cohort [6].

### Statistical methods

SPSS software was used for the statistical analyses (IBM SPSS Statistics, version 22, 2013, IBM Corp., Armonk, NY, USA). Relapse, SMN, death in remission and death after relapse were defined as events. The EFS and overall survival (OS) after relapse were estimated using the Kaplan–Meier method and differences in outcome between patients groups were tested using the Log Rank method. Comparisons of patient characteristics between subgroups were performed using Fisher’s exact test, Chi square test or ANOVA where appropriate. Prognostic factors after relapse were evaluated by using the Cox multivariate, proportional regression analysis. The significance level was set to p = 0.05.

## Results

### Long term survival of the patients included in the HIT-SIOP-PNET4 study

**Fig. 1:**
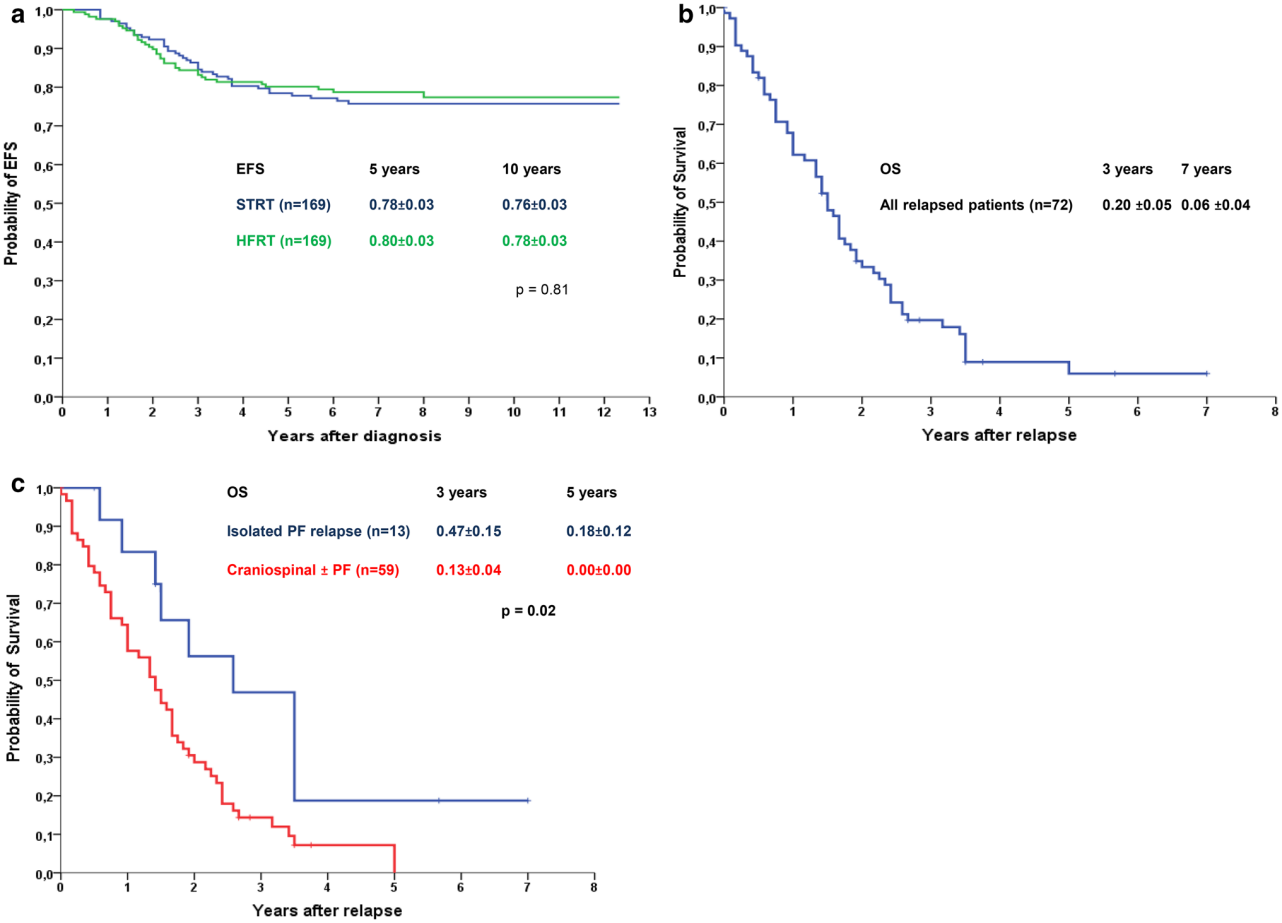
**a** Long-term probability of event free survival (EFS) of the two randomised treatment arms of HIT-SIOP-PNET4 at 5 and 10 years after primary diagnosis (Kaplan–Meier, Log Rank test). **b** Probability of overall survival (OS) after first relapse in the HIT-SIOP-PNET4 cohort (Kaplan–Meier). **c** Probability of overall survival (OS) after first relapse in relation to site of relapse (Kaplan–Meier, Log Rank test)

At 10 years from diagnosis, EFS and OS for all the 338 patients were 76 ± 2 % and 78 ± 2 % respectively. The EFS for the two treatment arms continued to show no significant difference; HFRT 78 ± 3 %, STRT 76 ± 3 % (Fig. 1a). At the time when data collection was halted, 72/338 patients (21 %) had suffered a relapse and three patients a SMN (see below). One patient died in CCR1. Four patients were lost to follow up.

In order to make our long term results comparable with the COG A9961 study [1], and only for this particular calculation, two adjustments were made: 31 patients with large residual tumours >1.5 cm^2^ at diagnosis were excluded as well as another 21 patients who had local but not central review of postoperative MRI performed which could allow for disseminated disease being missed. The adjusted EFS and OS at 10 years, were 79 ± 3 % and 81 ± 2 % (n = 286). All other results below are based on the total patient group.

### Diagnosis of relapse

MRI scans diagnosing relapse were centrally reviewed in 29/72 cases (40 %). Information on the reason for performing the MRI that demonstrated relapse was available in 65 patients (90 %). Forty-five relapses (69 %) were detected on surveillance MRI. For the remaining 20 patients whose relapse presented symptomatically, the most common symptoms were headache and/or vomiting (n = 11), followed by back pain (n = 5) and loss of specific neurological function (n = 3). The patients presenting with symptoms had a significantly shorter survival after relapse than those detected by surveillance MRI p < 0.01, but the OS (after primary diagnosis) did not differ p = 0.21 (Log Rank test).

### Relapse site and timing

The 72 relapses occurred at a mean/median of 30/26 months from diagnosis; range 2–95 months. All patients had complete radiological staging with cranial and spinal MRI at relapse. Metastatic CNS relapse with or without involvement of the PF was the most common relapse site; 59/72 patients (82 %), at a mean/median of 28/26 months after diagnosis. The majority of metastatic relapses, 47/59 (80 %), were multifocal, including cases with leptomeningeal dissemination. In the remaining 20 % there was a solitary metastatic relapse in the spine (n = 7) or brain (n = 5) (Table 1). Eighteen relapses (25 %) were confined exclusively to the spine, and occurred at a mean/median of 30/26 months from diagnosis; range 9–65 months. Twelve of these were diagnosed by surveillance MRI. Eight (44 %) of the MRI scans showing only spinal recurrence were centrally reviewed. There was information on CSF cytology in 61/73 patients. Twenty-one (34 %) had malignant cells in CSF at relapse, always in combination with other tumour manifestations. An isolated local relapse in PF was seen in 13 patients (18 %) after a mean/median of 38/35 months. Late relapse i.e. >5 years from initial diagnosis, occurred in 6 patients (8 %). Five of these were isolated relapses in PF (n = 3) or spine (n = 2). There was no difference in relapse distribution (isolated PF vs not isolated PF) between early and late relapses, although there was a trend for more late, isolated PF relapses (p = 0.07, Fisher’s exact test). Late relapses were diagnosed by surveillance MRI in 2/6 and due to symptoms in 2/6, (2/6 no data). Neither the timing nor the site of relapse differed between the two randomised treatment arms. Furthermore, we compared patients who received ≤4 chemotherapy courses vs patients who received >4 (max 8) courses in the primary tumour treatment, and found no significant difference in the number of relapses between these two groups, (p = 0.398, Fisher’s exact test).

**Table 1:** Treatment of relapse according to site

Site of relapse	n (%)	Surgery (%)	RT (%)	Chemotherapy (%)	HDSCR (%)
Isolated PF	13 (18)	6/13 (46)	2/13 (15)	11/11^b^ (100)	6/12^a^ (50)
Isolated ST or spinal	12 (17)	5/12 (38)	5/12 (38)	8/8^c^ (100)	3/12 (25)
Multiple craniospinal^e^ ± PF	47 (65)	7/47 (14)	9/47 (19)	41/41^d^ (100)	6/47 (13)
Total	72 (100)	18/72 (25)	16/72 (22)	60/66 (90)	15/72 (21)

*HDSCR* high dose chemotherapy with autologous stem cell rescue, *PF* posterior fossa, *RT* radiotherapy, *ST* supratentorial

Information missing in ^a^1 patient, ^b^2 patients, ^c^4 patients, ^d^6 patients, ^e^includes leptomeningeal dissemination

### Relapse in relation to histology and biology at primary diagnosis

Histology at primary diagnosis (classic 57, desmoplastic 10, large cell/anaplastic 5) was not related to time in CCR1, (p = 0.56, ANOVA), localisation, (p = 0.97, Chi square) or survival after relapse, (overall p = 0.32, Log Rank test). Nuclear β-catenin IHC status at diagnosis was known in 58/72 patients (14 % positive, 8/58 tumours assessed) and *CTNNB1* mutation status in 55 (5 % mutated, 3/55), *MYC*/*MYCN* status in 33/72 patients (9 % *MYCN* amplified, 3/33, no *MYC* amplified), and chromosome 17 imbalances/diploid background in 32/72 patients (ch17(im)/diploid(cen); 31 %, 10/32) [6]. Neither nuclear β-catenin status nor Chr 17 status had any significant impact on time in CCR1, (p = 0.50/0.51 respectively, ANOVA), localisation (p = 0.61/0.07, Chi square) or survival after relapse, (p = 0.60/0.46, Log Rank test). Eight tumours showing nuclear beta-catenin accumulation relapsed, and in 4/8 of these other unfavourable risk factors were present (Table 2). Six of these relapses were previously reported [6], two more patients have relapsed since that report. Notably, none of eight relapsed *WNT*-activated MBs is alive in remission (7 DOD). Only seventeen patients underwent surgery at relapse. Therefore, an analysis of outcome with respect to histology and biology on tumour material taken at relapse was not feasible.

**Table 2:** Patients with *WNT*-activated tumours (positive nuclear beta-catenin IHC) and relapse

ID	CTNNB1 mutation analysis	Other risk factors	Relapse site	CCR1 (months)	Relapse treatment		Survival after 1st relapse (months)	Status
					R1	R2 (R3)		
W1	Pos	a	PF	22	HDSCT (CBDCA, VP-16)	TMZ + IT maphosphamide (HITSKK-92, Mtx/Depocyte)	42	DOD
W2	Pos	No	Met (ST)	40	No	No (TMZ, VP-16, GEMOX)	45	AWT
W3	Pos	a, d	Met (Spinal)	18	TMZ	TMZ	17	DOD
W4	Neg	a, d, r	Met (CSF, spinal)	31	IT VP-16 + surgery + RT	TMZ	16	DOD
W5	Neg	No	PF, Met	53	Oral VP-16	N/A	2	DOD
W6	No data	d	Met (Spinal)	54	Trophosphamide + VP-16 + RT	TMZ + IT VP-16	29	DOD
W7	No data	No	PF	67	Surgery + CBDCA, VP-16 + HDSCR (TT, VP-16)	No	18	DOD
W8	No data	No	PF, Met (CSF)	16	No data		29	DOD

Four patients had risk factors for poor prognosis at primary diagnosis; a = age >16 years at diagnosis, d = delayed RT start, r = residual (primary) tumour >1.5 cm^2^. All tumours were of classic histological subtype except case W5 (desmoplastic MB). Relapse treatment at first (R1) and subsequent relapses (R2, R3)

*AWT* alive with tumour, *CBDCA* carboplatin, *CCR1* time in continuous complete remission after primary diagnosis, *DOD* dead of disease, *GEMOX* gemcitabine + oxaliplatin, *CSF* cerebrospinal fluid, *HDSCT* high dose chemotherapy with autologous stem cell rescue, *IT* intrathecal, *Met* metastatic relapse in CNS (location), *Mtx* methotrexate, *PF* posterior fossa, *RT* radiotherapy, *ST* supratentorial, *TMZ* temozolomide, *TT* thiotepa, *VP*-*16* etoposide

### Relapse treatment

In four patients no relapse treatment was given, (no treatment data available in one patient). All remaining 67 patients received treatment. The combination of treatments comparing those given to isolated PF vs all other relapses is shown in Table 1. Surgery was performed in 25 % with radical surgery in less than half of the procedures, RT in 22 %, conventional chemotherapy in 90 % and HDSCR in 21 % of all relapsed patients (Table 1). Surgery and HDSCR were significantly more often used (p = 0.027) in isolated PF relapses. Fifteen patients received re-treatment with focal RT (20-45 Gy), most often to spinal metastases (n = 10), supratentorial localised relapse (n = 3) and PF relapse (n = 2). The most common chemotherapy drug was temozolomide alone or in combination with other drugs. Carboplatin-based combinations were also common (Table 3).

**Table 3:** Chemotherapy and intrathecal treatment at relapse

Chemotherapy drugs	Number of patients		
	All patients	Isolated PF-relapses	Metastatic relapses
Temozolomide	24	2	22
Carboplatin + etoposide ± cyclophosphamide	15	3	12
Cyclophosphamide	5	0	5
Trophosphamide + etoposide	3	0	3
Temozolomide + irinotecan	4	2	2
Gemcitabine + oxaliplatin	2	0	2
Other combinations	3	1	2
Drugs not specified	5	3	2
No chemotherapy	7	1	6
No data	4	1	3
Intrathecal chemotherapy drugs	17		
Etoposide	7		
Methotrexate	6		
Cytarabine	3		
Other	1		
No intrathecal chemotherapy	49		
No data	6		

Information on the use of intrathecal chemotherapy was available in 66 patients with 17 (26 %) receiving either etoposide (n = 7), methotrexate (n = 6), Depocyte™ (liposomal cytarabine) (n = 3) and one unknown. Concordant information on CSF cytology and intrathecal chemotherapy in 53 patients showed that 9/21 (43 %) patients with CSF positivity received intrathecal treatment as did 5/32 (16 %) patients without malignant cells in CSF.

### Survival after relapse

The mean/median survival time after relapse was 23/18 months (Fig. 1b). The OS after relapse, was 20 % (± 5 %) at 3 years, and 6 % (± 4 %) at 5 years after relapse. The OS after isolated PF relapse was significantly higher compared to all other relapses (p = 0.02) (Fig. 1c). In Cox multivariate analysis, surgery (p < 0.01) and isolated PF relapse (p < 0.01) were associated with longer survival after relapse unlike treatment with RT (p = 0.10) or HDSCR (p = 0.44). When the data base was frozen, nine patients were still alive. A detailed description of the survivors is shown in Table 4.

**Table 4:** Biological data, relapse localisation, treatment and survival time in all patients still alive when database was frozen

ID	β-Catenin status	MYC / MYCN ampl	Ch17	CCR1 (mo)	Relapse site	Surgery	RT	Chemotherapy	IT	HDSCR	OS (mo)
1	Neg	No	No	30	PF local	GTR	No	CBDCA, VP-16	MTX	No	114
2	Neg	No	No	27	PF local	GTR^a^	No	TMZ	No	No	95
3^b^	ICH + Mut+	No	No	42	ST local	No	No	TMZ, VP-16, gemcitabine, oxaliplatin^c^	No	No	87
4	No data	No data	No data	65	Spine multiple	PR	No	CBDCA, CPM	No	No	107
5	Neg	No data	No data	44	Spine multiple	No	Yes	CBDCA, CPM, VP-16	No	Yes	78
6	Neg	No	No	51	ST local	Yes	Yes	TMZ	No	No	82
7	Neg	No data	No data	75	PF + spine	Yes	No	No	No data	No	98
8	Neg	No	No	95	PF local	GTR	No	Yes	No	Yes	113
9	Neg	No	No	72	PF	Yes	No	Yes	No data	Yes	78

Histology: classic MB (9/9). One patient was randomised to HFRT at primary treatment (ID 8), the rest to STRT

*CBDCA* carboplatin, *Ch17* chromosome 17 imbalances/diploid background, *CCR1* time in continuous complete remission after primary diagnosis (months), *CPM* cyclophosphamide, *GTR* gross total resection, *HDSCR* high dose chemotherapy with autologous stem cell rescue, *HFRT* hyperfractionated radiotherapy, *ICH*+ immunohistochemistry for nuclear β-catenin positive, *IT* intrathecal chemotherapy, *mo* months, *Mut*+ *CTNNB1* mutation positive, *OS* overall survival after primary diagnosis (months), *PF* posterior fossa, *PR* partial resection, *RT* radiotherapy, *ST* supratentorial, *STRT* standard (conventional) radiotherapy, *TMZ* temozolomide, *VP*-*16* etoposide

^a^Surgery performed at 3rd local relapse

^b^Same as case W2 in Table 2

^c^Chemotherapy initiated after observation period, when relapsed tumour progressed

### Second malignant neoplasms (SMN)

Three SMNs were reported as primary events namely: a pontine anaplastic astrocytoma, a PF glioblastoma, and an abdominal rhabdoid tumour in a child with Li-Fraumeni syndrome occurring 61, 55, and 35 months after primary diagnosis respectively. All three patients have died. Review of the Li-Fraumeni case confirmed the original (desmoplastic) MB diagnosis (INI-1 positive, SHH-activated with p53 alteration).

## Discussion

The primary aim of the HIT-SIOP-PNET4 study was to compare two radiotherapy protocols. It was hypothesised that the HFRT regimen used would be superior for survival without causing more late effects. With a median follow up time of 7.8 years, the estimated OS at 10 years, remains not significantly different in the two treatment arms. Since some patients with standard risk MB are known to relapse later than 5 years after diagnosis (in this study 8 % of the patients), a long follow up is necessary to definitely accept the null hypothesis.

The Children’s Oncology Group (COG) A9961 RCT Study, published in 2012, comparing two different chemotherapy protocols while giving the same conventional RT as in our study, reported a 10-year OS of 81.3 ± 2.1 % [1] which is very similar to our adjusted OS of 81 ± 2 %. The time to relapse, median 23 months, and the amount of disseminated relapses with a tendency to occur earlier than local PF relapses, is also in line with the COG study [1]. Consequently, the two largest studies of standard risk MB, including almost 700 patients, have produced very similar long-term results albeit using slightly different treatment regimes. It is noteworthy that in both studies, patients who were not optimally staged at primary diagnosis (due to incomplete/poor quality MRIs or no central review of MRIs), or had excess residual tumour (>1.5 cm^2^) on review, had worse outcome [2, 8].

A relevant question today is whether the MB subgroup could explain the pattern and timing of relapse [1]. Ramaswamy et al. showed that the different MB subgroups had different relapse patterns, with more local relapses in patients with Sonic Hedgehog (SHH) tumours compared to Group 3 and 4 tumours, which tended to relapse with metastases [9]. Furthermore, patients with Group 4 tumours were shown to survive longer after relapse, compared to the other subgroups [9]. In this study, only the *WNT*-activated subgroup could be prospectively delineated, and insufficient tumour material remained to do further retrospective subgrouping. It is well known that tumours showing β-catenin nuclear accumulation (IHC) are prognostically favourable [10]. Indeed only eight patients with relapse showed this biomarker, however, the timing and pattern of relapse for *WNT*-activated tumours did not differ from the non-*WNT* tumours. The prognosis after relapse is poor also for this group, and frontline de-escalation of therapy should only be done within trials, as salvage options are limited.

The majority of relapses were found on surveillance imaging when the patients had not yet developed signs or symptoms of relapse and seemingly these patients had a longer survival after relapse. This confirms previous findings but is probably due to the effect of lead time and length time bias as eventually almost all patients died due to progressive disease [3, 11]. The authors agree with others that with respect to survival, the benefit of early discovery of relapse by surveillance MRI remains unclear [12, 13], but could be justified in the perspective of possible inclusion in a relapse study, e.g. an early drug development trial. In that context, a spinal MRI should be considered, since 17 % of relapses were both asymptomatic and confined only to the spine. The high frequency of exclusively spinal recurrences (25 %) contrasts to a smaller study by Bartels et al., where 12/24 relapses had a spinal component but no exclusively spinal relapses were found [12]. Admittedly, this discrepancy could to some extent be explained by the lack of consistent central review of MRI scans in the present study, but since 8/18 (44 %) of cases with exclusive spinal relapse had a central review of the MRI scan, it seems that spinal relapses (leptomeningeal or solid) without concurrent cranial involvement do occur.

We found a relatively low incidence of SMNs, with only three cases reported compared to the COG A9961 study from which fifteen cases were reported albeit with a few years longer follow up. We should thus expect more SMNs and it appears that this is becoming a major problem affecting outcome as MB patients survive longer [1]. However, some SMNs may have gone undetected due to the low number of tumour biopsies at relapse 17/72 (24 %), in the present study.

The poor outcome after MB relapse for patients previously treated with radiotherapy is in line with other reports [3, 4, 11, 14, 15]. With regard to the use of HDSCR, there was hope that this approach would be of significant benefit in relapsed malignant brain tumours, and single institution reports showed encouraging results [16]. However, some studies that reported survival from the time of HDSCR may particularly have over-estimated the benefit of HDSCR-based strategies to the total population of relapsing patients. As noted in both the UK and German studies discussed below, a significant proportion of patients with chemo-resistant disease may not reach HDSCR despite an initial treatment plan to include HDSCR with curative intent.

There have been only limited national studies specifically for relapsed MB that aimed to investigate treatment that may provide long-term disease control. In the UK CCLG relapsed PNET study (2000–2007), at a median follow-up of 7.4 years, only three MB patients were alive, (5 year OS of 8.2 %) [15]. Similarly in 2014, Bode et al. published the results of the German HIT-REZ-97 trial [4]. This national study tested a non-randomised but stratified relapse protocol using either intensive chemotherapy +/- HDSCR as a potentially curative therapy or oral chemotherapy as a palliative option. Survival was poor with only 2/72 patients alive and in CCR at the time of the report. As of September 2015, these two patients are alive and in CCR 155 and 145 months after first relapse (G Fleischhack—personal communication). HDSCR was associated with severe toxicity with a treatment related mortality rate of 7.4 %. The poor results of a HDSCR-based approach have also been shown in recent small institutionally based studies [17, 18]. To date there has been no randomised trial evaluating the role of HDSCR in MB relapse in a multimodal therapy approach and in comparison with conventional chemotherapy, metronomic chemotherapy, targeted therapy or anti-angiogenic therapy.

As noted in this study, oral palliative chemotherapy with, for example, temozolomide or etoposide was frequently employed and may provide time-limited disease control [14]. There has been recent interest in low intensity multi-agent drug combinations with antiangiogenic effect often referred to as ‘metronomic chemotherapy’. In Europe, such therapy is being investigated in studies such as MEMMAT and COMBAT [19, 20], with reports showing a degree of promise with respect to disease control.

The role of intrathecal therapy at relapse is difficult to evaluate. It was not used to its full extent in this study considering that less than half of the patients with malignant cells in CSF at relapse received this treatment. However, the non-randomised German HIT relapse study which involved the use of intraventricular etoposide prior and simultaneously to the conventional systemic chemotherapy showed short-term disease stabilisation in a relevant number of patients but did not achieve higher survival [21]. Re-irradiation, although not a factor for prolonged survival in the present study, has previously been shown to be of benefit, and considering the high frequency of metastatic relapses in MB, craniospinal re-irradiation has been suggested as a therapeutic option worth exploring, although this requires careful balancing against the risk of side effects [22].

A limitation of this study is the lack of information on tumour biology at relapse. This area is now being actively investigated. For example, Wang et al. demonstrated subgroup stability across the primary and the metastatic compartments in newly diagnosed MB [23]. Ramaswamy et al. showed subgroup stability between tumour at diagnosis and relapse in MB [9] and Hill et al. recently undertook a comprehensive investigation of 29 relapsed MB that confirmed this finding [5]. Importantly, Hill et al. also demonstrated significant changes in biological characteristics of tissue at relapse compared to diagnosis. In particular, *MYC* family (*MYC, MYCN*) gene amplifications and *TP53* pathway defects commonly emerged in combination at relapse and predicted rapid progression to death [5]. Another limitation is that only 40 % of the relapses were confirmed by central review of MRI scans, and also the diversity of relapse treatments.

The strength of this study is the long-time follow up of standard risk MB in children treated according to the same protocol in over one hundred European institutions. This would indicate an outcome not biased by treatment at few or tertiary centres. The report shows the outcome of relapse treatment without the use of a common protocol and gives a survival curve based on a variety of treatments to which further studies could be compared.

The optimal approach to treating relapsed MB in previously irradiated children remains in doubt. The question is still whether it is possible to identify a subgroup of patients in whom a curative approach is justified, and if this is possible, what therapy should be applied. Certainly, in cases where relapse is localised, surgical resection is appropriate. Surgery has added value with regard to provide material to enhance our understanding of this disease and to exclude a SMN. It appears clear however, that the vast majority of relapsed patients cannot be cured and in most patients exploration of new drugs in early phase trials is appropriate, preferably guided by biopsies of the relapsed tumour, and not only the primary tumour.

## References

[1] Packer RJ, Zhou T, Holmes E, Vezina G, Gajjar A (2012). Survival and secondary tumors in children with medulloblastoma receiving radiotherapy and adjuvant chemotherapy: results of Children’s Oncology Group trial A9961. Neuro Oncol.

[2] Lannering B, Rutkowski S, Doz F, Pizer B, Gustafsson G, Navajas A, Massimino M, Reddingius R, Benesch M, Carrie C, Taylor R, Gandola L, Bjork-Eriksson T, Giralt J, Oldenburger F, Pietsch T, Figarella-Branger D, Robson K, Forni M, Clifford SC, Warmuth-Metz M, von Hoff K, Faldum A, Mosseri V, Kortmann R (2012). Hyperfractionated versus conventional radiotherapy followed by chemotherapy in standard-risk medulloblastoma: results from the randomized multicenter HIT-SIOP PNET 4 trial. J Clin Oncol.

[3] Bouffet E, Doz F, Demaille MC, Tron P, Roche H, Plantaz D, Thyss A, Stephan JL, Lejars O, Sariban E, Buclon M, Zucker JM, Brunat-Mentigny M, Bernard JL, Gentet JC (1998). Improving survival in recurrent medulloblastoma: earlier detection, better treatment or still an impasse?. Br J Cancer.

[4] Bode U, Zimmermann M, Moser O, Rutkowski S, Warmuth-Metz M, Pietsch T, Kortmann RD, Faldum A, Fleischhack G (2014). Treatment of recurrent primitive neuroectodermal tumors (PNET) in children and adolescents with high-dose chemotherapy (HDC) and stem cell support: results of the HITREZ 97 multicentre trial. J Neurooncol.

[5] Hill RM, Kuijper S, Lindsey JC, Petrie K, Schwalbe EC, Barker K, Boult JK, Williamson D, Ahmad Z, Hallsworth A, Ryan SL, Poon E, Robinson SP, Ruddle R, Raynaud FI, Howell L, Kwok C, Joshi A, Nicholson SL, Crosier S, Ellison DW, Wharton SB, Robson K, Michalski A, Hargrave D, Jacques TS, Pizer B, Bailey S, Swartling FJ, Weiss WA, Chesler L, Clifford SC (2015). Combined MYC and P53 defects emerge at medulloblastoma relapse and define rapidly progressive, therapeutically targetable disease. Cancer Cell.

[6] Clifford SC, Lannering B, Schwalbe EC, Hicks D, O’Toole K, Nicholson SL, Goschzik T, Zur Muhlen A, Figarella-Branger D, Doz F, Rutkowski S, Gustafsson G, Pietsch T (2015). Biomarker-driven stratification of disease-risk in non-metastatic medulloblastoma: results from the multi-center HIT-SIOP-PNET4 clinical trial. Oncotarget.

[7] Ryan SL, Schwalbe EC, Cole M, Lu Y, Lusher ME, Megahed H, O’Toole K, Nicholson SL, Bognar L, Garami M, Hauser P, Korshunov A, Pfister SM, Williamson D, Taylor RE, Ellison DW, Bailey S, Clifford SC (2012). MYC family amplification and clinical risk-factors interact to predict an extremely poor prognosis in childhood medulloblastoma. Acta Neuropathol.

[8] Packer RJ, Gajjar A, Vezina G, Rorke-Adams L, Burger PC, Robertson PL, Bayer L, LaFond D, Donahue BR, Marymont MH, Muraszko K, Langston J, Sposto R (2006). Phase III study of craniospinal radiation therapy followed by adjuvant chemotherapy for newly diagnosed average-risk medulloblastoma. J Clin Oncol.

[9] Ramaswamy V, Remke M, Bouffet E, Faria CC, Perreault S, Cho YJ, Shih DJ, Luu B, Dubuc AM, Northcott PA, Schuller U, Gururangan S, McLendon R, Bigner D, Fouladi M, Ligon KL, Pomeroy SL, Dunn S, Triscott J, Jabado N, Fontebasso A, Jones DT, Kool M, Karajannis MA, Gardner SL, Zagzag D, Nunes S, Pimentel J, Mora J, Lipp E, Walter AW, Ryzhova M, Zheludkova O, Kumirova E, Alshami J, Croul SE, Rutka JT, Hawkins C, Tabori U, Codispoti KE, Packer RJ, Pfister SM, Korshunov A, Taylor MD (2013). Recurrence patterns across medulloblastoma subgroups: an integrated clinical and molecular analysis. Lancet Oncol.

[10] Ellison DW, Onilude OE, Lindsey JC, Lusher ME, Weston CL, Taylor RE, Pearson AD, Clifford SC (2005). beta-Catenin status predicts a favorable outcome in childhood medulloblastoma: the United Kingdom Children’s Cancer Study Group Brain Tumour Committee. J Clin Oncol.

[11] Torres CF, Rebsamen S, Silber JH, Sutton LN, Bilaniuk LT, Zimmerman RA, Goldwein JW, Phillips PC, Lange BJ (1994). Surveillance scanning of children with medulloblastoma. N Engl J Med.

[12] Bartels U, Shroff M, Sung L, Dag-Ellams U, Laperriere N, Rutka J, Bouffet E (2006). Role of spinal MRI in the follow-up of children treated for medulloblastoma. Cancer.

[13] Saunders DE, Hayward RD, Phipps KP, Chong WK, Wade AM (2003). Surveillance neuroimaging of intracranial medulloblastoma in children: how effective, how often, and for how long?. J Neurosurg.

[14] Cefalo G, Massimino M, Ruggiero A, Barone G, Ridola V, Spreafico F, Potepan P, Abate ME, Mascarin M, Garre ML, Perilongo G, Madon E, Colosimo C, Riccardi R (2014). Temozolomide is an active agent in children with recurrent medulloblastoma/primitive neuroectodermal tumor: an Italian multi-institutional phase II trial.. Neuro Oncol.

[15] Pizer B, Donachie PH, Robinson K, Taylor RE, Michalski A, Punt J, Ellison DW, Picton S (2011). Treatment of recurrent central nervous system primitive neuroectodermal tumours in children and adolescents: results of a Children’s Cancer and Leukaemia Group study. Eur J Cancer.

[16] Graham ML, Herndon JE, Casey JR, Chaffee S, Ciocci GH, Krischer JP, Kurtzberg J, Laughlin MJ, Longee DC, Olson JF, Paleologus N, Pennington CN, Friedman HS (1997). High-dose chemotherapy with autologous stem-cell rescue in patients with recurrent and high-risk pediatric brain tumors. J Clin Oncol.

[17] Gururangan S, Krauser J, Watral MA, Driscoll T, Larrier N, Reardon DA, Rich JN, Quinn JA, Vredenburgh JJ, A, McLendon RE, Fuchs H, Kurtzberg J, Friedman HS, Desjardins (2008). Efficacy of high-dose chemotherapy or standard salvage therapy in patients with recurrent medulloblastoma. Neuro Oncol.

[18] Massimino M, Gandola L, Spreafico F, Biassoni V, Luksch R, Collini P, Solero CN, Simonetti F, Pignoli E, Cefalo G, Poggi G, Modena P, Mariani L, Potepan P, Podda M, Casanova M, Pecori E, Acerno S, Ferrari A, Terenziani M, Meazza C, Polastri D, Ravagnani F, Fossati-Bellani F (2009). No salvage using high-dose chemotherapy plus/minus reirradiation for relapsing previously irradiated medulloblastoma. Int J Radiat Oncol Biol Phys.

[19] Sterba J, Pavelka Z, Andre N, Ventruba J, Skotakova J, Bajciova V, Bronisova D, Dubska L, Valik D (2010). Second complete remission of relapsed medulloblastoma induced by metronomic chemotherapy. Pediatr Blood Cancer.

[20] Peyrl A, Chocholous M, Kieran MW, Azizi AA, Prucker C, Czech T, Dieckmann K, Schmook MT, Haberler C, Leiss U, Slavc I (2012). Antiangiogenic metronomic therapy for children with recurrent embryonal brain tumors. Pediatr Blood Cancer.

[21] Tippelt S, Mikasch R, Warmuth-Metz M, Pietsch T, Hilger RA, Kwiecien R, Faldum A, Rutkowski S, Bode U, Siegler N, Fleischhack G (2014). CT-002. Intraventricular therapy with etoposide in recurrent medulloblastomas, pineoblastomas, CNS-PNETs and ependymomas—final results of a phase II study. Neuro Oncol.

[22] Wetmore C, Herington D, Lin T, Onar-Thomas A, Gajjar A, Merchant TE (2014). Reirradiation of recurrent medulloblastoma: does clinical benefit outweigh risk for toxicity?. Cancer.

[23] Wang X, Dubuc AM, Ramaswamy V, Mack S, Gendoo DM, Remke M, Wu X, Garzia L, Luu B, Cavalli F, Peacock J, Lopez B, Skowron P, Zagzag D, Lyden D, Hoffman C, Cho YJ, Eberhart C, MacDonald T, Li XN, Van Meter T, Northcott PA, Haibe-Kains B, Hawkins C, Rutka JT, Bouffet E, Pfister SM, Korshunov A, Taylor MD (2015). Medulloblastoma subgroups remain stable across primary and metastatic compartments. Acta Neuropathol.

